# A new remarkable species of *Alloscorpiops* Vachon, 1980 from Myanmar (Burma) (Scorpiones, Scorpiopidae)

**DOI:** 10.3897/zookeys.775.24248

**Published:** 2018-07-17

**Authors:** Wilson R. Lourenço, Ondřej Košulič

**Affiliations:** 1 Muséum national d’Histoire naturelle, Sorbonne Universités, Institut de Systématique, Evolution, Biodiversité (ISYEB), UMR7205-CNRS, MNHN, UPMC, EPHE, CP 53, 57 rue Cuvier, 75005 Paris, France; 2 Department of Forest Protection and Wildlife Management, Faculty of Forestry and Wood Technology, Mendel University in Brno, Zemědělská 3, Brno, Czech Republic

**Keywords:** *Alloscorpiops*, biodiversity, Burma, new species, Scorpion, Scorpiopidae, southeast Asia

## Abstract

Among the genera of the family Scorpiopidae Kraepelin, 1905 *Alloscorpiops* remains yet rather discrete. New species were added to this genus only recently, increasing its number from two to six. Therefore, species of *Alloscorpiops* can be considered rare and uncommonly collected. One particular new species, *Alloscorpiops viktoriae* **sp. n.**, is described based on two females and one pre-adult male collected from the northern part of central Myanmar (Burma). The new species presents most features exhibited by scorpions of the genus *Alloscorpiops*, but it is characterised by a moderate to small size, very strongly marked granulation, and a particular trichobothrial pattern. Aspects of the ecology and distribution of the new species are discussed and compared with those of other species of genus *Alloscorpiops*.

## Introduction

As already outlined by Lourenço and Pham (2015a, b) in a revision of the genus *Scorpiops* (Lourenço and Pham 2015a, b), Vachon (1980) described three new subgenera, *Alloscorpiops*, *Euscorpiops*, and *Neoscorpiops*, in addition to the nominotypical subgenus Scorpiops. *Alloscorpiops* was defined based on an important ‘majorante’ neobothriotaxy with 10-12 ventral trichobothria on the surface of pedipalp chela-hand, whereas the other subgenera presented only four trichobothria. Vachon (1980) assigned two species to this subgenus: Scorpiops (Alloscorpiops) anthracinus Simon, 1887 (as type species of the subgenus) and Scorpiops (Alloscorpiops) lindstroemii Thorell, 1889.

Stockwell (1989), in an unpublished thesis dissertation, proposed raising all the subgenera within the family Scorpiopidae to the rank of genera; however, his proposition could not be validated since his dissertation was never published. Finally, Lourenço (1998) confirmed this decision. The four subgenera were elevated to generic rank and the monotypic genera *Parascorpiops* Banks, 1928 and *Dasyscorpiops* Vachon, 1974 were added, thus bringing the total number of genera to six.

In the present note, one additional new species belonging to the genus *Alloscorpiops* is described from the region of Magway in the northern part of central Myanmar. Specimens were collected in an open sandy riverbed, which is not a common habitat type for this group of scorpions, as all known species are usually found in dry or humid tropical forest ecosystems (e.g., Kovařík 2013; Kovařík et al. 2013; Lourenço 2017). Moreover, currently all *Alloscorpiops* species occupy an area around central to southern Thailand or the Mekong region in eastern Indochina. Therefore, the new *Alloscorpiops* species extends its area of distribution, with central Myanmar now forming its northernmost point. It may represent yet another endemic element in the fauna of this country.

## Materials and methods

Illustrations and measurements were produced using a Wild M5 stereo-microscope with a drawing tube and an ocular micrometre. Measurements follow Stahnke (1970) and are given in mm. Trichobothrial notations follow Vachon (1974, 1980), morphological terminology mostly follows Vachon (1952) and Hjelle (1990), and chelicerae dentition follows Vachon (1963). Locality data were recorded using portable GPS units (Garmin Oregon 450). The map background was downloaded from Free Vector Maps platform system and modified in Adobe Illustrator CS3 and Photoshop CS4.

### Present composition of the genus *Alloscorpiops* Vachon, 1980

*Alloscorpiops anthracinus* (Simon, 1887), Myanmar

*Alloscorpiops lindstroemii* (Thorell, 1889), Myanmar

*Alloscorpiops calmonti* Lourenço, 2013, Laos

*Alloscorpiops citadelle* Kovařík, 2013, Thailand

*Alloscorpiops wongpromi* Kovařík, Soleglad & Košulič, 2013, Laos, Thailand

*Alloscorpiops troglodytes* Lourenço & Pham, 2015, Vietnam

*Alloscorpiops viktoriae* sp. n., central Myanmar (this study)

## Taxonomic treatment

### Family Scorpiopidae Kraepelin, 1905

#### Genus *Alloscorpiops* Vachon, 1980

##### Alloscorpiops (Alloscorpiops) viktoriae sp. n.

Taxon classificationAnimaliaScorpionesScorpiopidae

http://zoobank.org/FF6038DF-4F70-4B05-B5BA-92D443AA5445

Figs 1–2 , 3–5 , 6–12

###### Diagnosis.

The new species shows several of the characteristics already defined for the genus *Alloscorpiops* (Vachon, 1980). General colouration reddish brown to dark brown. Global size moderate to small in relation to other species of the genus; adult female with 50.9 mm in total length and a very strong overall granulation. The new species is also characterised by the trichobothrial patterns of some ‘territories’ or series. Femur with three trichobothria, ***d***, ***i*** and ***e***. Patella with the trichobothria ***d1*** and ***d2*** on the dorsal surface; ***i*** on the internal surface, 15-17 ***V*** on the ventral surface and only 22 trichobothria on the external surface (6 ***et***, 7 ***est***, 2 ***em***, 2 ***esb***, 5 ***eb***). Chela-hand with an unusual trichobothrial number on the ***V*** series of 8-9 on the ventral surface, ***Dt*** on the dorsal surface, ***Db*** on the external surface, ***ib*** and ***it*** on the internal surface, five ***Et***, ***Est***, ***Esb*** and three trichobothria in the ***Eb*** series on the external surface. The annular ring is strongly marked. Pectines with 8-9 teeth in females and 8-8 in the only known male; fulcra reduced.

**Figures 1–2.**
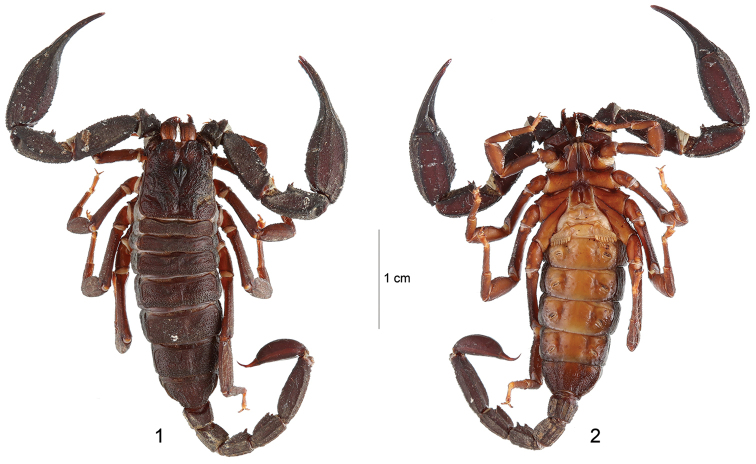
*Alloscorpiops viktoriae* sp. n. Female holotype. Habitus. Dorsal and ventral aspects.

###### Material.

Myanmar (Burma), Magway region, Kyakhtu District, River Stream, Sandy habitat, GPS 21°27’36”N; 94°16’24”E, 398 m a.s.l., 29/I/2016 (O. Košulič). Female holotype (RS-9122) and male-juvenile paratype (RS-9123) deposited in the Muséum national d’Histoire naturelle, Paris. Female paratype (VS-55342) deposited in the Mendel University of Brno, Czech Republic.

###### Etymology.

The new species is named after a young lady, Viktorie Košuličová, the daughter of O. Košulič. Coincidently, the new species was also found in the region close to the most impressive peak of Central Myanmar, Mt. Victoria.

###### Description.

The general coloration is reddish brown to dark brown. Carapace and tergites reddish brown. Metasomal segments brown to dark brown; telson reddish brown; base of aculeus yellow and tip slightly reddish. Chelicerae yellow with intense variegated brownish spots; teeth reddish. Pedipalps dark brown; granulations on chela fingers almost reddish. Legs reddish brown. Venter reddish to reddish yellow; genital operculum and pectines yellow.

**Figures 3–5.**
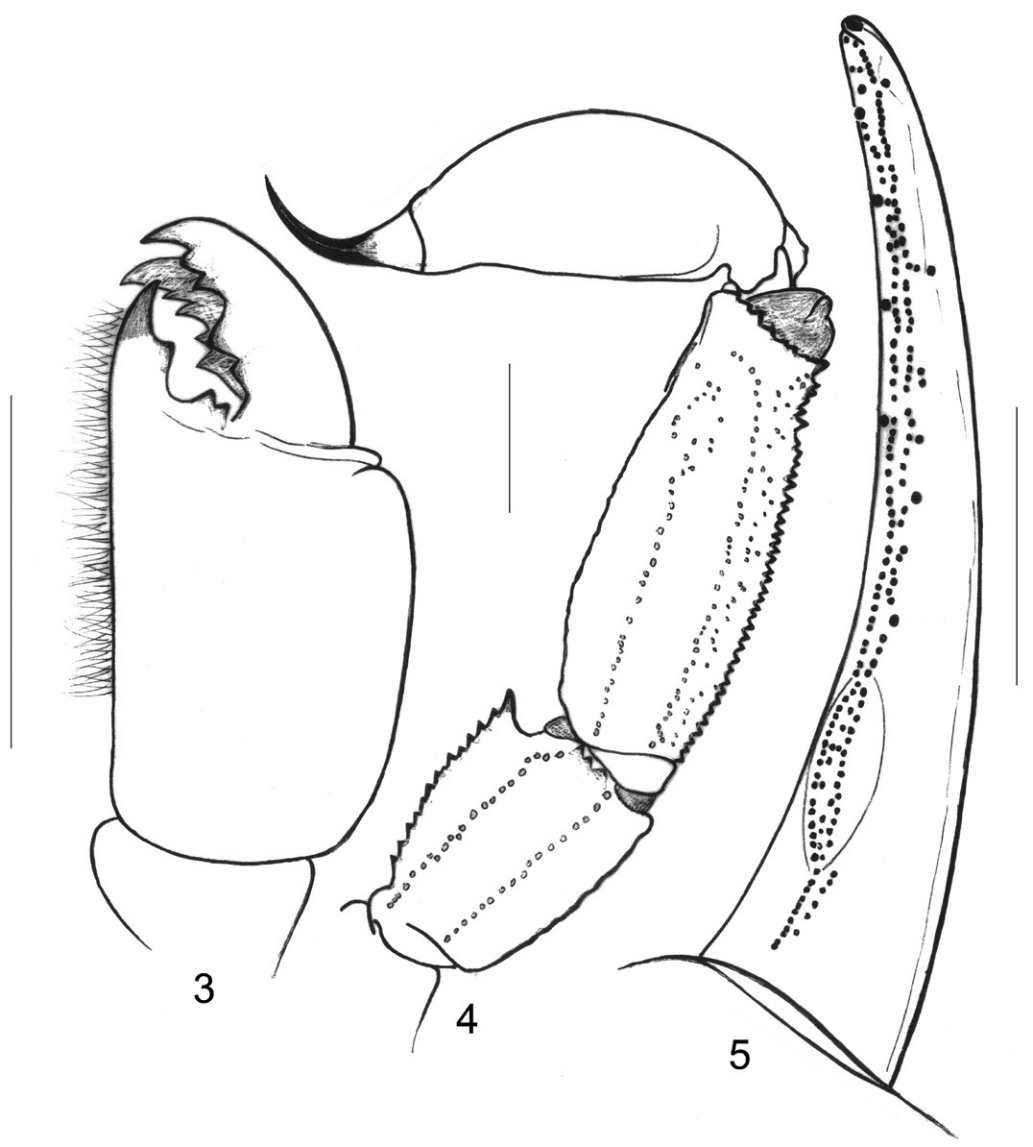
*Alloscorpiops viktoriae* sp. n. Female holotype. **3** Chelicera, dorsal aspect **4** Metasomal segments VI–V and telson, lateral aspect **5** Cutting edge of movable finger with rows of granules. Scale bars 2 mm.

**Figures 6–12.**
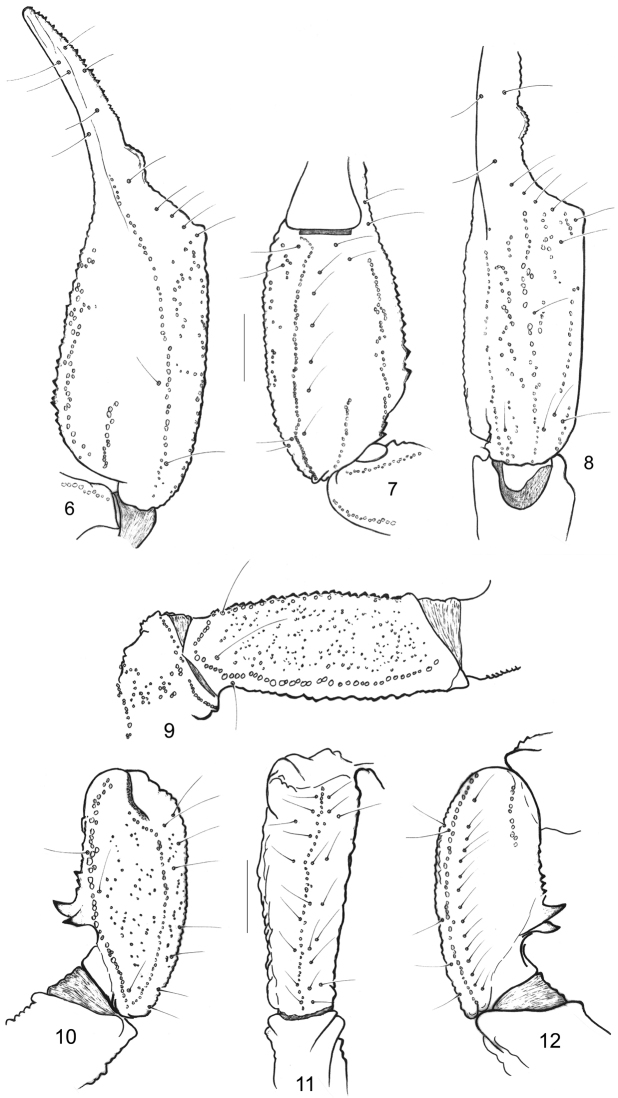
*Alloscorpiops viktoriae* sp. n. Female holotype. Trichobothrial pattern. **6–8** Chela, dorso-external, ventral and internal aspects **9** Femur, dorsal aspect **10–12** Patella, dorsal, external and ventral aspects. Scale bars 2 mm.

*Morphology*. Carapace strongly granular, furrows moderately to very deep. Median eyes anterior to the centre of carapace; three pairs of lateral eyes, the third pair only slightly smaller than the first two. Sternum pentagonal, longer than wide. Genital operculum formed by two semi-oval plates in female. Tergites strongly granulated; VII with four moderately marked carinae. Pectinal tooth count 8-8 (9-9) in females, 8-8 in male; fulcra reduced. Sternites smooth and shiny; VII with four weak carinae and some granulations. Metasomal segments I and II wider than long; segments III to V longer than wide; 10-8-8-8-7 carinae present on segments I–V, strongly marked; dorsal carinae on segments I-IV, with strongly marked posterior spinoid granules on segments III-IV; metasomal tegument moderately to strongly granulated; ventral carina on segment V with weak spinoid granules. Telson vesicle almost smooth, with some isolated granulations. Pedipalps: femur with dorsal internal, dorsal external, ventral internal and ventral external carinae moderately to strongly marked; tegument moderately granular. Patella with dorsal internal, ventral internal, dorsal external, ventral external and external carinae strongly marked; several spinoid granules present on internal aspect, two of which are very conspicuous; the interno-ventral being larger than the interno-dorsal granule; tegument moderately granular. Chela with dorsal marginal, external secondary, ventral internal and ventral carinae moderately to strongly marked; other carinae moderately marked; tegument granulated dorsally and ventrally. Chelal fingers with two longitudinal series of granules, almost fused, and a few inner and outer accessory granules. Chelicerae dentition as illustrated in Figure 3; four/five teeth on ventro-internal face of movable finger. Trichobothriotaxy type **C**, as presented in Figs 6–12.

###### Relationships.

Although geographically closer to the others species of *Alloscorpiops* described from Myanmar (Burma) the new species shows some affinities with *Alloscorpiops troglodytes* from Vietnam, in particular by the reduced number of trichobothria in some territories. Both species can, however, be readily distinguished by the structure of their tegument, which is weakly granular, almost smooth in *A. troglodytes* and strongly granular in *A. viktoriae* sp. n. Besides this, they differ in their overall size, pattern of pigmentation, and general morphology (see also the following key).

Morphometric values (in mm) of female holotype. Total length (including telson) 50.9. Carapace: length 8.0; anterior width 4.1; posterior width 7.9. Mesosoma length 17.8. Metasomal segment I: length 2.2, width 3.1; II: length 2.4, width 2.7; III: length 3.2, width 2.6; IV: length 3.6, width 2.4; V: length 6.3, width 2.2, depth 2.2. Telson length 7.4. Vesicle: width 2.2, depth 2.1. Pedipalp: femur length 7.9, width 3.0; patella length 7.2, width 3.3; chela length 15.9, width 4.4, depth 4.3; movable finger length 7.7.

##### Simplified key to the species of *Alloscorpiops*

**Table:** 

1	Chela of pedipalp with 3 trichobothria on the **Eb** series	**2**
–	Chela of pedipalp with 5 trichobothria on the **Eb** series	**Alloscorpiops (Laoscorpiops) calmonti**
2	Chela of pedipalp with 10–13 ventral trichobothria; patella with 15–22 ventral trichobothria	**4**
–	Chela of pedipalp with 8–9 ventral trichobothria; patella with 14–17 ventral trichobothria	**3**
3	Tegument of carapace and tergites almost smooth	***Alloscorpiops troglodytes***
–	Tegument of carapace and tergites strongly granulated	***Alloscorpiops viktoriae* sp. n.**
4	Patella of pedipalp with 15–16 ventral and 23–25 external trichobothria	**5**
–	Patella of pedipalp with 19–21 ventral and 29–37 external trichobothria	**6**
5	Patella of pedipalp with 16 ventral and 23 external trichobothria	***Alloscorpiops anthracinus***
–	Patella of pedipalp with 15 ventral and 25 external trichobothria	***Alloscorpiops lindstroemii***
6	Patella of pedipalp with 19–21 ventral and 29–34 external trichobothria	***Alloscorpiops citadelle***
–	Patella of pedipalp with 21–22 ventral and 33–37 external trichobothria	***Alloscorpiops wongpromi***

##### Type locality and habitat of *Alloscorpiops viktoriae* sp. n.

**Figure 13.**
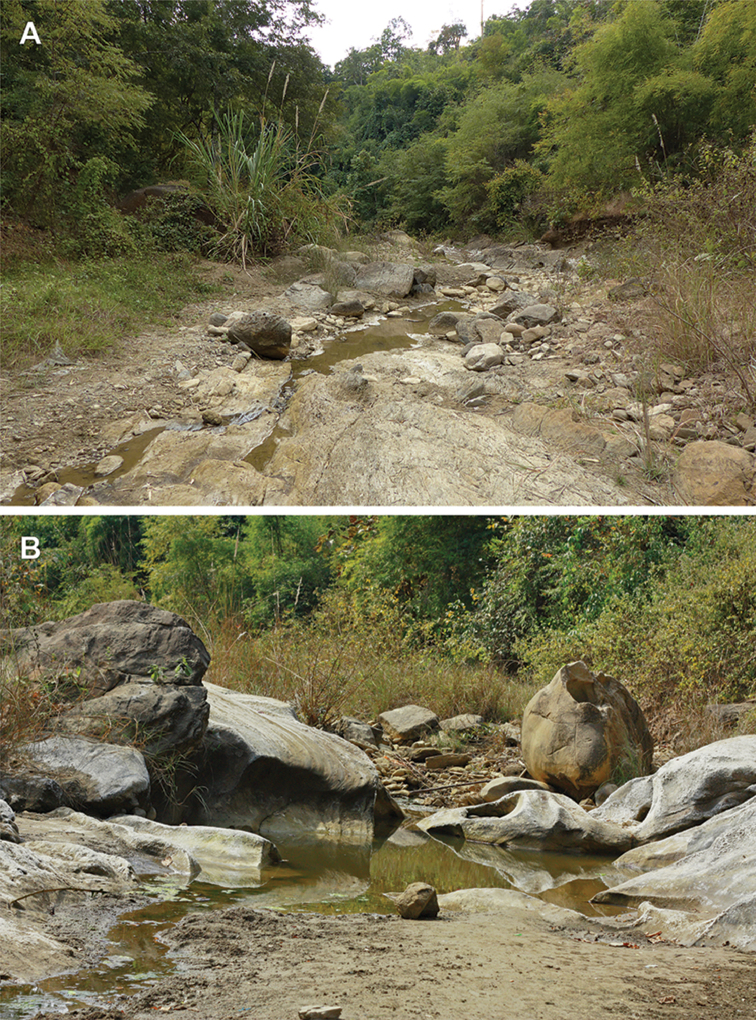
Type locality of *Alloscorpiops viktoriae* sp. n. in Magway region of Central Myanmar. **A** Overall view on natural habitat of the new species **B** Detail view on the same habitat. All specimens were found under stones located directly in humid riverbed. Photographs by Ondřej Košulič (**A**); Šárka Mašová (**B**).

The new species *A. viktoriae* sp. n. was found in Magway region in the northern part of central Myanmar along the border with Chin State. This region is situated on the eastern slopes of the Chin Hills of Arakan Mountains, which includes a large area of tropical and subtropical broadleaf forests (Leimgruber et al. 2005). The Arakan Mountains act as a barrier to the southwestern monsoon and thus shield central Myanmar, making their eastern slopes much drier (Wu et al. 2004). The hilly landscape area of the type locality was located at a rather lower elevation, approximately 300–400 m a.s.l. The area is significantly disturbed by anthropogenic influence and covered mainly by fragmented dry dipterocarp and bamboo forests, agricultural fields, and uncultivated rocky-sandy habitats with scattered woody vegetation. The specimens were found hiding under stones in sandy riparian habitats along a small water stream (Figure 13). The stream was surrounded by dry dipterocarp and bamboo forests with moderately open canopy coverage in a very dry condition (Figure 13). Several specimens of *Lychas mucronatus* (Fabricius, 1798) were also sympatrically found. No other species of scorpions were observed during the field trip to this area. We suggest that specimens of *A. viktoriae* sp. n. moved from very dry conditions of dipterocarp and bamboo forests to the stream in search of higher humidity during the peak of the dry season. Such a pattern can also be found in other arthropod groups during long-term dry conditions in tropical forests (Murphy and Lugo 1986).

##### Distribution and ecological affinities of species belonging to the genus *Alloscorpiops*

**Figure 14.**
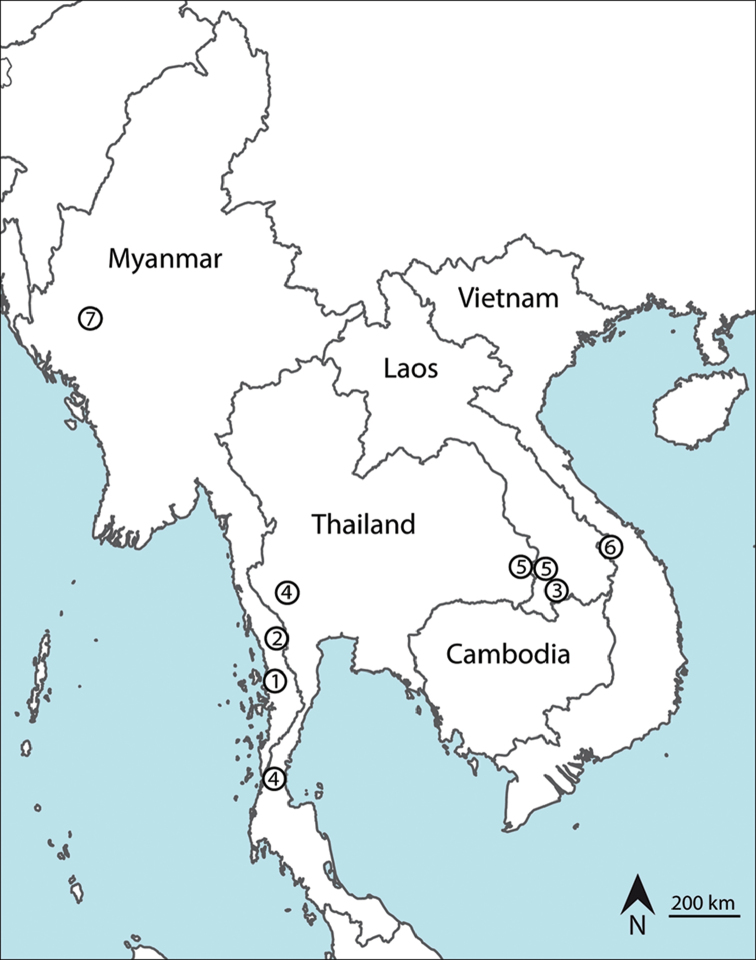
Map of southeast Asia showing the known distribution of the species belonging to the genus *Alloscorpiops*: *Alloscorpiops anthracinus* (**1**), *Alloscorpiops lindstroemii* (**2**), *Alloscorpiops calmonti* (**3**), *Alloscorpiops citadelle* (**4**), *Alloscorpiops wongpromi* (**5**), *Alloscorpiops troglodytes* (**6**) and *Alloscorpiops viktoriae* sp. n. (**7**).

Until now, all species from the genus *Alloscorpiops* were distributed between 15° and 8° of geographical latitude and 98° to 107° of geographical longitude. This area is located from central Thailand to the eastern region of Indochina along southern Laos and central Vietnam to southern Thailand where the southernmost distribution of this genus is (Figure 14). Therefore, our finding of *A. viktoriae* sp. n. indicates the northernmost location of this genus and significantly extends the region of distribution of *Alloscorpiops* further to the central of Myanmar (Figure 14). We suppose that more findings may be discovered in suitable locations in southeast Asia; however, it also seems that these scorpions are very localised and due to their life strategy and good hiding abilities they may be easily overlooked in tropical ecosystems.

With the exception of *A. troglodytes*, which was found in a cave habitat in a limestone area of central Vietnam (Lourenço 2015), all species were usually discovered in lowland dry dipterocarps or bamboo forests (Kovařík et al. 2013; Lourenço 2013) or dry evergreen tropical forests (Simon 1887; Thorell 1889). One questionable situation has been observed for *A. citadelle* Kovařík, 2013. This species was described from a type locality in Khlong Phanom in southern Thailand. The region is characterised by the presence of humid tropical rain forests influenced from the south by the Malesia bioregion. Furthermore, Kovařík (2013) also described this species based on one juvenile paratype from Sai Yok in central Thailand. However, this area is influenced by different biogeographical regions (mainly from Indochina) with the presence of forest types (e.g., dry evergreen and deciduous forests) and ecological characteristics quite distinct from those in southern Thailand (Knight and Holloway 1990). Therefore, we suggest that Kovařík (2013) probably misidentified this juvenile paratype as *A. citadelle.* The specimen from central Thailand could in fact be associated with another species of *Alloscorpiops* such as *A. anthracinus* or *A. lindstroemii*, which naturally occur in forest ecosystems of this region or with some other possibly new species from this genus.

In general, most *Alloscorpiops* species have been collected and observed from similar microhabitat conditions sharing ecological strategies similar to other groups from the family Scorpiopidae (e.g., Qi et al. 2005, Kovařík et al. 2015). Specimens of *Alloscorpiops* can be found and observed in the already mentioned habitats during the evening or at night in a sit-and-wait position resting inverted on overhanging surfaces of rock or soil walls. Some of them may occupy more protected places in fissures of cracked rock walls. When disturbed, the scorpions usually escape and hide deeper in the rock fissures or soil burrows (Kovařík et al. 2013). They can also be found under dead wood (Lourenço 2013), in crevices in caves (Lourenço 2015) or under stones such as *A. viktoriae* sp. n.

In conclusion, it can be suggested that *A. viktoriae* sp. n. represents a remarkable species of scorpion for Myanmar and its finding significantly extends the distribution range of this southeast-Asian endemic genus further to the north from its original area. Presumably more species from this group will be found in suitable habitats across southeast Asia.

## Supplementary Material

XML Treatment for Alloscorpiops (Alloscorpiops) viktoriae
